# Cancer Predisposition Genes in Cancer-Free Families

**DOI:** 10.3390/cancers12102770

**Published:** 2020-09-27

**Authors:** Guoqiao Zheng, Calogerina Catalano, Obul Reddy Bandapalli, Nagarajan Paramasivam, Subhayan Chattopadhyay, Matthias Schlesner, Rolf Sijmons, Akseli Hemminki, Dagmara Dymerska, Jan Lubinski, Kari Hemminki, Asta Försti

**Affiliations:** 1 Division of Molecular Genetic Epidemiology, German Cancer Research Center (DKFZ), Im Neuenheimer Feld 580, D-69120 Heidelberg, Germany; guoqiao.zheng@med.lu.se (G.Z.); Calogerina.Catalano@med.uni-heidelberg.de (C.C.); o.bandapalli@kitz-heidelberg.de (O.R.B.); subhayan.chattopadhyay@med.lu.se (S.C.); k.hemminki@dkfz-heidelberg.de (K.H.); 2 Department of Internal Medicine V, University of Heidelberg, 69120 Heidelberg, Germany; 3 Hopp Children’s Cancer Center (KiTZ), 69120 Heidelberg, Germany; 4 Division of Pediatric Neurooncology, German Cancer Research Center (DKFZ), German Cancer Consortium (DKTK), 69120 Heidelberg, Germany; 5 Medical Faculty, University of Heidelberg, 69120 Heidelberg, Germany; 6 Computational Oncology, Molecular Diagnostics Program, National Center for Tumor Diseases (NCT), 69120 Heidelberg, Germany; n.paramasivam@dkfz-heidelberg.de; 7 Bioinformatics and Omics Data Analytics, German Cancer Research Center (DKFZ), 69120 Heidelberg, Germany; m.schlesner@Dkfz-Heidelberg.de; 8 Department of Genetics, University Medical Center Groningen, University of Groningen, 9700 RB Groningen, The Netherlands; r.h.sijmons@umcg.nl; 9 Cancer Gene Therapy Group, Translational Immunology Research Program, University of Helsinki, 00290 Helsinki, Finland; akseli.hemminki@helsinki.fi; 10 Comprehensive Cancer Center, Helsinki University Hospital, 00290 Helsinki, Finland; 11 Hereditary Cancer Center, Department of Genetics and Pathology, Pomeranian Medical University, 70-111 Szczecin, Poland; dymerska@pum.edu.pl (D.D.); lubinski@pum.edu.pl (J.L.); 12 Faculty of Medicine and Biomedical Center in Pilsen, Charles University in Prague, 30605 Pilsen, Czech Republic; 13 Division of Cancer Epidemiology, German Cancer Research Center (DKFZ), 69120 Heidelberg, Germany

**Keywords:** predisposing genes, high-risk genes, polygenic risk, random environment

## Abstract

**Simple Summary:**

Familial clustering of cancer and identification of high- and low-risk cancer predisposition gene variants implicate that there are families that are at a high to moderate excess risk of cancer. We wanted to test genetically whether there are families protected from cancer. We whole-genome sequenced 51 elderly individuals without any personal or family history of cancer. We identified less high-risk loss-of-function variants in known and suggested cancer predisposition genes in these cancer-free individuals than in the general population. However, our results for low-risk variants were not conclusive. Our study suggests that random environmental causes of cancer are so dominant that a clear demarcation of cancer-free populations using genetic data may not be feasible. However, carrier identification of and counseling about prevalent high-risk cancer predisposition genes is useful.

**Abstract:**

Familial clustering, twin concordance, and identification of high- and low-penetrance cancer predisposition variants support the idea that there are families that are at a high to moderate excess risk of cancer. To what extent there may be families that are protected from cancer is unknown. We wanted to test genetically whether cancer-free families share fewer breast, colorectal, and prostate cancer risk alleles than the population at large. We addressed this question by whole-genome sequencing (WGS) of 51 elderly cancer-free individuals whose numerous (ca. 1000) family members were found to be cancer-free (‘cancer-free families’, CFFs) based on face-to-face interviews. The average coverage of the 51 samples in the WGS was 42x. We compared cancer risk allele frequencies in cancer-free individuals with those in the general population available in public databases. The CFF members had fewer loss-of-function variants in suggested cancer predisposition genes compared to the ExAC data, and for high-risk cancer predisposition genes, no pathogenic variants were found in CFFs. For common low-penetrance breast, colorectal, and prostate cancer risk alleles, the results were not conclusive. The results suggest that, in line with twin and family studies, random environmental causes are so dominant that a clear demarcation of cancer-free populations using genetic data may not be feasible.

## 1. Introduction

Familial cancer (i.e., two or more first-degree relatives diagnosed with the same cancer) accounts for 25% of prostate cancer, 16% of breast cancer, and 15% of colorectal cancer [1]. For rarer cancers, the proportions go down to about 2%. These proportions are much lower than twin estimates on the heritability of various cancers [2,3]. This may imply, among various explanations, that population genetics is characterized by common genes and polygenes of low penetrance, which would rarely aggregate in families [1,4,5]. Germline genetics of cancer, as presently known, depends on the type of cancer. For common cancers, such as breast and colorectal cancers, mutations in high-risk predisposition genes *BRCA1/2* and mismatch repair genes are rare, accounting for a small proportion of the particular cancers (depending on population, approximately 1%) [6,7,8]. A number of other high-risk genes are known, but mutations in these are even rarer [9]. In addition, numerous and ever-increasing numbers of low-risk gene variants have been described for these cancers [10,11]. For other common cancers, including prostate and lung cancers, high-penetrance genes are rarer but also for these cancers numerous low-risk variants have been identified [8,9]. Combined, the high and low-risk variants explain a small proportion of the known familial risk and even less about the heritability estimated on twins.

A three-generation analysis in the Swedish Family-Cancer Database found that 16% of cancers were diagnosed in the third generation individuals whose two older generations were cancer-free, yet the relative risk (RR) of 0.9 showed no dramatic protection [12]. Recently, a whole-genome sequencing (WGS) project among 2570 healthy elderly within the Medical Genome Reference Bank in Australia reported fewer disease-associated common and rare germline variants compared to both cancer cases and the gnomAD and UK biobank cohorts [13]. Here, we identified 51 elderly cancer-free index persons (born in the 1920s or 1930s) whose siblings and relatives in one or two older and younger generations were cancer-free. We used WGS to test genetically whether cancer-free families (CFFs) share fewer cancer risk alleles than the population at large. We estimated that the CFFs, from which an index individual was sequenced, covered a total of 1000 cancer-free individuals.

## 2. Results

**Figure 1:**
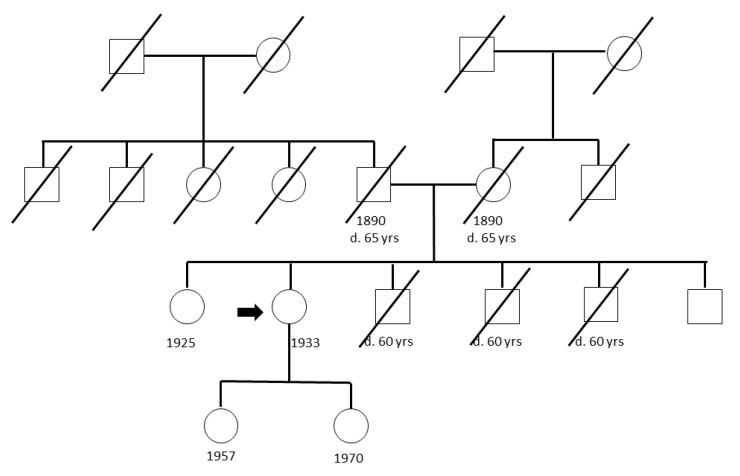
Pedigree of one cancer-free family with the index case indicated by an arrow.

A pedigree of a CFF is shown in Figure 1 pointing out the 80-year-old index person with an arrow. In this, as in other families, the siblings as well as the individuals in the older generation(s) were either alive or had died due to reason other than cancer. The index case of each family was whole genome sequenced with an average coverage of 42x.

### 2.1. Low-Risk Variants

The analysis of the low-risk alleles included a total of 106 single-nucleotide polymorphisms (SNPs) for breast cancer, 81 SNPs for colorectal cancer, and 105 SNPs for prostate cancer identified in five large meta-analyses of whole-genome association studies (GWASs) [8,14,15,16]. The genotypes of these SNPs were determined from the WGS data of the CFFs based on the position of the SNP in the reference human genome (build GRCh37, assembly hs37d5). Table 1 compares the risk allele frequencies of the low-risk variants between the CFFs and the data from the gnomAD database. Only SNPs with nominally significant *p*-value < 0.05 in the analysis are shown. For breast cancer, risk allele frequencies for five SNPs were lower and for two SNPs higher than for the gnomAD data. The only variant for colorectal cancer was rarer in CFFs than in gnomAD and for prostate cancer risk allele frequencies for four SNPs were lower and for six SNPs higher in CFFs than in gnomAD.

The total number of risk alleles was calculated for each individual and their distribution is shown in Supplementary Figure S1. The aggregation of the low-risk alleles in CFF individuals were tested against the 1000 Genomes data for which individual genotype data were available (Table 2). Based on the total number of risk alleles, the individuals were divided in quartiles with approximately equal numbers of individuals in each quartile in the 1000 Genomes population. Compared to the 1000 Genomes population, the proportion of CFF individuals decreased with the increasing number of breast cancer risk alleles, for colorectal cancer there was no change, and for prostate cancer, the proportion of CFF individuals increased with the increasing number of risk alleles.

### 2.2. Suggested Cancer Predisposition Genes

Next, we calculated the probability of an individual in the CFFs and the ExAC population of carrying potentially pathogenic variants in suggested cancer predisposition genes obtained from two different sources [17,18] (Table 3). Pathogenicity was evaluated using the criteria of our in-house developed Familial Cancer Variant Prioritization Pipeline version 2 (FCVPPv2) [19]. We extracted all variants in these genes from the WGS data of the 51 CFF individuals and from the ExAC data. After filtering the variants according to the criteria of the FCVPPv2, 54 non-synonymous variants in 50 genes, and two loss-of-function variants in two genes were classified as potentially pathogenic in CFFs among the 723 genes reported by Wei et al. [18], while 23,419 non-synonymous variants in 367 genes and 3675 loss-of-function variants in 482 genes passed the filters in the ExAC population. Among the 114 cancer predisposition genes reported by Rahman [17], 18 non-synonymous variants in 14 genes and no loss-of-function variants were classified as potentially pathogenic in CFFs, while 5619 non-synonymous variants in 70 genes and 791 loss-of-function variants in 81 genes passed the filters in ExAC. The probability of carrying a non-synonymous variant in genes reported both by Wei et al. and Rahman was higher in CFFs than in ExAC, while the probability of a CFF individual to carry a loss-of-function variant was lower in genes of the Wei et al. list and no loss-of-function variants in genes of the Rahman list were detected.

### 2.3. High-Risk Breast, Colorectal, and Prostate Cancer Predisposition Genes

We searched the WGS data of the CFF individuals for missense and loss-of function variants within the known high-risk genes *BRCA1* and *BRCA2* for breast cancer, *APC*, *MLH1*, *MSH2*, *MSH6*, *MUTYH*, and *PMS2* for colorectal cancer and *HOXB13* for prostate cancer. In Table 4, we list the high-risk gene variants with MAF < 0.001 found in the CFF individuals and report the number of the missense and loss-of-function variants in ExAC and the probability of an ExAC individual to carry at least one pathogenic/likely pathogenic variant. For the CFF variants, the scaled PHRED-like Combined Annotation-Dependent Depletion CADD score, number of positive conservation (three tools) and deleteriousness (10 tools) predictions, and the ClinVar significance are shown. In the ExAC population, 1692 missense or loss-of-function variants were reported of which 98 were classified as pathogenic/likely pathogenic by ClinVar. In CFF, each of the listed 12 missense or loss-of-function variants occurred only once and none of them were classified as pathogenic. No variants were found for *BRCA1* and *HOXB13*. ClinVar predicted all the CFF variants to be benign or likely benign, except that the *MUTYH* variant was reported to be likely pathogenic. Of note, *MUTYH* is a recessive cancer predisposition gene, and cancer might arise if a person inherited another mutated allele.

## 3. Discussion

In Poland, some 25% of all deaths are due to cancer, which is close to the average in Europe as reported by the World Health Organization (WHO) (http://www.euro.who.int/en/health-topics/noncommunicable-diseases/cancer/data-and-statistics). All persons with a cancer diagnosis do not die of cancer, and we can assume that 35% of Poles have a cancer in their lifetime. This would imply that among fully aged families of 10 persons, less than 1% would be cancer-free. Thus, such rare lucky families may exist by chance. However, although twin data suggest that cancer is largely a random environmental disease, family studies show that familial cancer is largely genetic, except for lung and cervical cancer with a large environmental component [2,3,20]. Therefore, the investigated 51 CFFs can be expected to show a reduced genetic predisposition to cancer.

The strongest evidence for lower predisposition to cancer in CFFs was that these individuals carried a lower frequency of loss-of-function alleles in suggested cancer predisposition genes but not of missense variants, as shown in Table 3. A relatively poor discrimination of missense variants for cancer risk has been reported earlier [4]. In the same vein, analysis of variants in high-risk cancer predisposition genes showed that the CFF population had 12 missense but no loss-of-function variants and none of these were classified as pathogenic by ClinVar, whereas in ExAC 98 of the 1692 identified variants were classified as pathogenic/likely pathogenic. The lack of loss-of-function variants in CFF was probably not surprising because only 51 individuals were tested. The 12 missense variants were benign as judged by the ClinVar significance, with one exception, *MUTYH*, which is a recessive cancer predisposition gene. Interestingly even though the ClinVar score indicated benign phenotype, the CADD scores were high (>20) for many of the variants.

The testing of low-risk variants did not give conclusive results. The frequencies of risk alleles in CFFs varied inconsistently around the frequencies in the gnomAD database (Table 1). Similarly, when CFF and the 1000 Genomes individuals were compared by the number of risk alleles, the proportion of CFF individuals decreased with the increasing number of breast cancer risk alleles, while an opposite trend was observed in prostate cancer. Data from GWASs on many cancers show that even collectively low-risk alleles explain a small proportion of the empirical familial risk [8,21]. It is known that usually low-risk alleles are moderately enriched in familial compared to sporadic cases, but even opposite results have been reported [22,23]. Improvement of risk prediction by adding a polygenetic risk score to prediction models that include the family history indicate only partial overlapping of these factors [24,25].

Overall, our results are concordant with the recent study on 2570 healthy elderly within the Medical Genome Reference Bank in Australia [13]. In that study, the participants did not have any personal history of cancer, cardiovascular disease, or dementia, while our study participants did not have any personal or family history of cancer in one or two older and younger generations that included around 1000 cancer-free individuals. A study of 51 individuals may not be impressive if one fails to recognize that all the index cases were over 70 years old and that these represent families each with an average of 20 elderly relatives none of whom were diagnosed with cancer. Unfortunately, the age of death data were not complete, although most of the deceased were known to have reached an age of late adulthood. Both studies reported fewer pathogenic/likely pathogenic variants in high-risk cancer predisposition genes, while we also showed that loss-of-function variants within suggested cancer predisposition genes were depleted in CFFs compared to the ExAC data. On the other hand, the Australian study showed depletion of common cancer risk alleles among the elderly population, which was not obvious in our study with 51 sequenced individuals.

It would also be interesting to search for genetic variants protecting against cancer, however, that would require large, well-characterized elderly population without any personal or family history of cancer. Even identification of cancer risk alleles is a challenging task, as shown by the GWASs on common cancers of breast, colorectum, and prostate in which over 100,000 individuals were genotyped [8,14,15,16].

Sample size was a limitation of the study even though the 51 sequenced individuals represented 1000 other individuals without known cancers. Unreported cancers may be another weakness of the study because information on cancer in relatives was based on anecdotal data. However, the family history data were collected by face-to-face interviews of individuals who had reported no cancer family history in questionnaires within a large population screening conducted earlier; thus, the data are likely to be more reliable than postal or telephone interviews. If the index persons were 80 years in 2010 their grandparents were 80 years at around 1950. Even though cancer was a known disease at that time, the incidence rates were earlier lower and thus the probability of being cancer-free was higher. Yet even currently well-functioning national cancer registries may miss up to 10% cancers, characterized by elderly patients and cancers, which may be diagnosed with debilitating comorbidities such as lung cancer [26]. Nevertheless, the overall cancer incidence in Poland is at a low European level, except for colorectal cancer, which is relatively common as shown in the Cancer Statistics-Specific Cancers by the European Union with data extracted in August 2020 (https://ec.europa.eu/eurostat/statistics-explained/pdfscache/39738.pdf). Another minor weakness is the likely genotypic stratification between the Polish population and the referent European populations. Overall, the European population is genetically very homogenous, although a more detailed analysis of population genetic structure using autosomal, Y-chromosome, and mitochondrial markers have shown closest Polish resemblance to the Eastern neighbors Russians, Belarusians, and Ukrainians, followed by Czechs, Slovaks, and Baltic populations [27,28,29,30]. To diminish bias related to population stratification and to exclude cancer patients from the analyses, we included only the non-Finnish European non-TCGA data from the ExAC and the gnomAD in our study. This may, however, have caused bias on our analyses, as the samples from CFFs and the ExAC and the gnomAD populations were sequenced on different platforms and the quality control was done separately. To avoid this bias, we used the quality filtering protocol, as suggested [31].

In conclusion, no striking genetic differences between the CFF and the unselected reference populations were detected. However, loss-of-function variants appeared to be at a lower frequency in CFF members, and for high-risk cancer genes, no loss-of-function variants were found in CFFs. The results appear to be consistent with the earlier finding from the Swedish Family-Cancer Database that the overall cancer risk is not markedly depressed (RR 0.9) if two previous generations are cancer-free because of random environmental and polygenic causes. They further agree with the notions suggesting that carrier identification of and counseling about prevalent high-risk cancer predisposition genes is useful, but the prospects of defining genetic basis for cancer protection may not be promising [32].

## 4. Materials and Methods 

### 4.1. Study Populations

The CFF group contained 51 individuals recruited by the Hereditary Cancer Center, Department of Genetics and Pathology, Pomeranian Medical University, Szczecin, Poland. Family histories were collected through face-to-face detailed interviews. An average interview took 20–30 min. In West-Pomeranian region of Poland, population screening was performed mainly in years 2000–2001, in which questionnaires about cancer family history were collected from about 1.25 million (~70%) of inhabitants. Persons with negative cancer family history were invited to outpatient clinics and asked to agree for recruitment to control group. In such a way, the group of about 1000 adult individuals was established. Persons selected for the present study were part of this control group. They all were over 70 years old at the time of recruitment.

Different reference groups were used to perform distinct statistical analyses; these included data from 64,603 (56,885 exome and 7718 genome individuals), 33,370, and 294 non-Finnish European (NFE) individuals extracted from the Genome Aggregation Database (gnomAD) (https://gnomad.broadinstitute.org/), the Exome Aggregation Consortium (ExAC) [33], and the 1000 Genomes database (https://www.internationalgenome.org/1000-genomes-browsers), respectively.

### 4.2. Ethics Statement

The ethical approval for this study design was obtained from the Bioethics Committee of the Pomeranian Medical Academy in Szczecin No: BN-001/174/05. Sample collection was performed following the guidelines proposed by this Committee. A written informed consent was signed by each participant in accordance with the Helsinki declaration.

### 4.3. Whole-Genome Sequencing

Whole-genome sequencing (WGS) of the cancer-free persons considered in the present study was performed in the Illumina X10 platform using DNA extracted from the blood samples. WGS was carried out as paired-end sequencing with a read length of 150 bp. Sequences were mapped to the reference human genome (build GRCh37, assembly hs37d5) using BWA mem (version 0.7.15) and duplicates were removed using Sambamba (version 0.1.19). Variants were called by using Platypus (version 0.8.1) and annotated using ANNOVAR [34], dbSNP [35], 1000 Genomes phase III [36], dbNSFP v3.0 [37], and ExAC [33], respectively. Variant filtering was carried out by considering a minimum of 5 reads coverage and a QUAL score higher than 20. To check for family relatedness, a pairwise comparison of variants among the cohort was performed. CFF, gnomAD, and ExAC data were filtered separately based on the criteria described in [31] and bases with a minimum of 10 reads coverage in at least 90% of samples were included in the analysis.

### 4.4. Low-Risk Variants

Five large recently published meta-analyses were used to collect single nucleotide polymorphisms (SNPs) predicted by genome-wide association studies (GWASs) to be associated with the risk of breast [8,15], colorectal [8,14], and prostate cancers [16] at the genome-wide significance level. SNPs with any of the following criteria were filtered out: (1) unspecified risk allele, (2) unspecified minor allele frequency (MAF) or MAF between 0.45 and 0.55, (3) effect size as odds ratio (OR) of the risk allele below 1.04, (4) only estrogen receptor (ER) status/histology-specific associations, (5) absence in the 1000 Genomes data, and (6) from two or more SNPs with pairwise linkage equilibrium (r^2^) higher than 0.8, only one was included. After filtering, 106, 81, and 105 SNPs for breast, colorectal, and prostate cancers, respectively, were used for further analyses. Logistic regression was performed to compare risk allele frequencies of the selected SNPs between CFFs and gnomAD data (used as the reference population). To account for the high number of tests, the significance level was adjusted using Bonferroni correction. In order to calculate a polygenic risk score, the logistic regression model was used to compare the number of risk alleles between CFFs and 294 non-Finnish European individuals from 1000 Genomes for which individual genotype data were available. The trend test was performed after dividing the individuals into quartiles based on the total number of risk alleles in individuals in 1000 Genomes and considering the groups as continuous variables.

### 4.5. Suggested Cancer Predisposition Genes

A comprehensive list of cancer predisposition genes was extracted from [17,18]. All missense and loss-of-function variants listed for each of these genes were downloaded from the ExAC data. Variants were filtered using the criteria of our in-house developed Familial Cancer Variant Prioritization Pipeline version 2 (FCVPPv2) [19]. MAF of 0.1% was used with respect to 1000 Genomes phase III, non-Finnish European non-TCGA ExAC data, and local datasets.

To select the top 10% of potentially deleterious variants in the human genome a scaled PHRED-like Combined Annotation-Dependent Depletion (CADD) score greater than 10 was applied [38]. Assuming that variants in genes intolerant to variation are likely to be deleterious, a screening for intolerance was performed; three different intolerance scores based on NHLBI-ESP6500 [39], ExAC datasets [33], and a local dataset with allele frequency data were considered. Additionally, the Z-score, developed by the ExAC consortium for missense and synonymous variants, was utilized [33].

To assess the evolutionary conservation of the variant position, three tools were used: Genomic Evolutionary Rate Profiling (GERP >2.0) [40], PhastCons (>0.3) [41], and Phylogenetic *p*-value (PhyloP ≥ 3.0) [42] with an inclusion of variants predicted to be located at a conserved genomic position by at least two tools. 

To evaluate the deleteriousness of the coding variants, prediction tools Sorting Intolerant from Tolerant (SIFT) [43], Polymorphism Phenotyping version-2 (PolyPhen-2) HDIV (HumDiv) [44], PolyPhen-v2 HVAR (HumVar) [44], Log ratio test (LRT) [45], MutationTaster [46], Mutation Assessor [47], Functional Analysis Through Hidden Markov Models (FATHMM) [48], MetaSVM [37], MetaLR [37], and Protein Variation Effect Analyzer (PROVEAN) [49] were used. Variants predicted to be deleterious by more than 50% of these tools were included in the further analyses.

To evaluate the probability that one individual from the CFFs (P_CFF_) and the ExAC (P_ExAC_) population, respectively, carries at least one potentially pathogenic variant, we used the method described by Castera et al. [50]. P_ExAC_ and P_CFF_ were calculated using the following formula: 1- the probability of one individual not carrying any pathogenic variants. Therefore, (1) in which (2) represented the probability that one ExAC individual from non-Finish European population carried the i^th^ variant among the k potentially pathogenic variants identified. OR was estimated by computing (3) and bias-corrected and accelerated (BCa) bootstrapping was performed to calculate 95% confidence interval (95%CI) of OR with 10,000 resampling [51].

(1) $$P_{\mathrm{ExAC}}=(1-\prod_{i=1}^{k}1-\frac{{\mathrm{AC}}_{\mathrm{NFEi}}-{\mathrm{Hom}}_{\mathrm{NFEi}}}{\left({\mathrm{AN}}_{\mathrm{NFEi}}/2\right)})$$

(2) $$\frac{{\mathrm{AC}}_{\mathrm{NFEi}}-{\mathrm{Hom}}_{\mathrm{NFEi}}}{\left({\mathrm{AN}}_{\mathrm{NFEi}}/2\right)}$$

(3) $$\frac{\mathrm{PCFF}(1-\mathrm{PExAC})}{(1-\mathrm{PCFF})\mathrm{PExAC}}$$

### 4.6. Variants in High-Risk Genes of Breast, Colorectal, and Prostate Cancer

We searched the WGS data of CFFs for missense and loss-of function variants within the known high-risk genes *BRCA1* and *BRCA2* for breast cancer, *APC*, *MLH1*, *MSH2*, *MSH6*, *MUTYH,* and *PMS2* for colorectal cancer and *HOXB13* for prostate cancer. The pathogenicity was evaluated using the ClinVar database (https://www.ncbi.nlm.nih.gov/clinvar/). We also screened the ExAC non-Finnish European data for missense and loss-of-function variants with MAF <0.001 that passed the ExAC QC filters. The probability that one individual of the ExAC carries at least one pathogenic/likely pathogenic variant reported in the ClinVar database was evaluated as described above.

All the statistical analyses were done using SAS version 9.4 and R version 3.5 (SAS Institute Inc., Cary, NC, USA).

## 5. Conclusions

Our whole-genome germline sequencing effort on 51 elderly cancer-free individuals whose numerous (ca. 1000) family members were found to be cancer-free implicated that the cancer-free family members had no pathogenic variants in high-risk breast, colorectal, and prostate cancer predisposition genes. They also had fewer loss-of-function variants in suggested cancer predisposition genes compared to the ExAC data. For common low-penetrance breast, colorectal, and prostate cancer risk alleles, the results were not conclusive. The results suggest that, in line with twin and family studies, random environmental causes are so dominant that a clear demarcation of cancer-free populations using genetic data may not be feasible.

## Figures and Tables

**Table 1:** Comparison of risk allele frequency between cancer-free families (CFFs) and gnomAD for breast, colorectal, and prostate cancers.

Cancer	SNPID	Gene	Risk Allele	Frequency		OR	95% CI		*p* ^1^
				GnomAD	CFF				
**BC**	rs10474352	*ARRDC3*	C	0.83	0.74	0.56	0.36	0.87	**0.0097**
	rs16886181	*MAP3K1*	C	0.17	0.08	0.41	0.20	0.85	**0.0165**
	rs206966	*RP1-166H1.2*	T	0.17	0.25	1.67	1.07	2.61	**0.0248**
	rs2992756	*KLHDC7A*	T	0.49	0.37	0.62	0.41	0.93	**0.0197**
	rs653465	*SLC4A7*	C	0.47	0.57	1.48	1.00	2.20	**0.0489**
	rs7072776	*DNAJC1*	A	0.28	0.19	0.58	0.35	0.96	**0.0348**
	rs889312	*MAP3K1*	C	0.29	0.18	0.53	0.32	0.89	**0.0154**
**CRC**	rs17816465	*GREM1*	A	0.20	0.10	0.43	0.23	0.84	**0.0125**
**PC**	rs10460109	*TSHZ1*	T	0.42	0.56	1.72	1.16	2.55	**0.0067**
	rs3850699	*TRIM8*	A	0.68	0.56	0.6	0.41	0.89	**0.0110**
	rs28607662	*TCF4*	C	0.09	0.17	1.96	1.16	3.31	**0.0118**
	rs2066827	*CDKN1B*	T	0.75	0.86	2.05	1.17	3.61	**0.0125**
	rs2680708	*RNF43*	G	0.60	0.48	0.62	0.42	0.91	**0.0153**
	rs33984059	*RFX7*	A	0.98	0.94	0.37	0.16	0.85	**0.0193**
	rs12155172	*LINC01162*	A	0.24	0.33	1.62	1.07	2.45	**0.0216**
	rs6465657	*LMTK2*	C	0.46	0.57	1.56	1.05	2.31	**0.0265**
	rs12543663	*PCAT1*	C	0.31	0.41	1.56	1.05	2.32	**0.0270**
	rs9364554	*SLC22A3*	T	0.27	0.19	0.60	0.37	1.00	**0.0478**

^1^*p*-value for Bonferroni adjusted significance level: breast cancer (BC), 0.05/106 = **0.0005**; colorectal cancer (CRC), 0.05/81 = **0.0006**; prostate cancer (PC), 0.05/105 = **0.0005;** OR: odds ratio; 95%CI: 95% confidence interval; SNPID, SNP identification number; *p*: *p*-value; bold values indicate statistical significance at *p* < 0.05.

**Table 2:** Combined effect of risk alleles related to breast, colorectal, and prostate cancers in cancer-free families (CFFs) and 1000 Genomes data.

Cancer	No. Risk Alleles	1000 Genomes No.	CFF No.	OR	95%CI		*p*
**BC**	≤87	73	19	1.00	-		-
	88–91	72	12	0.64	0.29	1.42	0.27
	92–96	77	11	0.55	0.24	1.23	0.15
	>96	72	9	0.48	0.20	1.13	0.09
	*p*-trend = 0.07						
**CRC**	≤71	75	13	1.00			
	72–76	90	13	0.83	0.36	1.91	0.67
	77–80	69	16	1.34	0.60	2.98	0.48
	>80	60	9	0.87	0.35	2.16	0.76
	*p*-trend = 0.88						
**PC**	≤89	91	10	1.00			
	90–93	64	10	1.42	0.56	3.62	0.46
	94–97	86	10	1.06	0.42	2.67	0.90
	>97	53	21	3.61	1.58	8.23	**0.0023**
	*p*-trend = **0.0055**						

BC: breast cancer; CRC: colorectal cancer; PC: prostate cancer; OR: odds ratio; 95%CI: 95% confidence interval; *p*: *p*-value.

**Table 3:** Comparison of the probability of carrying potentially pathogenic non-synonymous and loss of function (LoF) variants within cancer predisposition genes (CPGs) in cancer-free families (pCFF) and in the ExAC population (pExAC). Pathogenicity was evaluated using the criteria of our in-house developed Familial Cancer Variant Prioritization Pipeline version 2 (FCVPPv2).

Source of CPGs	CFF No. Variants	P CFF	ExAC No. Variants	P ExAC	OR	95%CI	
Wei [18] non-synonymous	54	67 %	23419	63 %	1.21	0.77	1.91
Wei [18] LoF	2	6 %	3675	15 %	0.35	0.00	0.53
Rahman [17] non-synonymous	18	31 %	5619	22 %	1.58	0.87	2.83
Rahman [17] LoF	0	0 %	791	4 %			

LoF: loss-of-function, stop gain/loss, splice-site, and frameshift indel variants; P: probability; OR: odds ratio; 95%CI: 95% confidence interval.

**Table 4:** List of variants in known high-risk genes in breast, colorectal, and prostate cancers found in cancer-free families (CFFs) with annotations. For the ExAc population, probability of carrying a pathogenic/likely pathogenic variant is shown.

Gene	Missense + LoF Variants in ExAC			Missense + LoF Variants in CFF								
	Total No.	No. Pathogenic	p ExAC ^1^	SNP ID	Chr	Position	Ref/Alt	Prevalence ExAC NFE	CADD	Positive Conservation Scores	Positive Prediction Tools	ClinVar Significance
*BRCA2*	691	40	0.112%	rs397507270	13	32907128	A/G	1.51 × 10^−5^	0.11	0	1	Likely benign/US
				rs56087561	13	32913562	A/C	3.65 × 10^−4^	24.1	2	5	Benign
				rs80358768	13	32913947	C/T	3.45 × 10^−4^	0.2	0	1	Benign
												
*APC*	481	2	0.003%	rs748940586	5	112178309	A/C	1.51 × 10^−5^	22.7	3	8	US
				No dbSNP	5	112178460	GTAT/G	.	21.8	.	.	-
												
*MLH1*	167	3	0.008%	rs41294980	3	37067306	G/A	1.18 × 10^−3^	7.3	1	0/4 ^2^	Benign
				rs63751225	3	37090075	T/C	1.80 × 10^−4^	22.1	3	4	US
												
*MSH2*	246	4	0.006%	rs116117580	2	47739533	G/A	1.99 × 10^−2^	0.003	0	1	Not provided
												
*MSH6*	359	8	0.017%	rs752887988	2	48010377	C/T	0	33	3	7	-
				rs267608075	2	48028282	A/T	1.83 × 10^−4^	13.0	3	5	Benign/US
												
*MUTYH*	174	12	0.079%	rs36053993	1	45797228	C/T	3.96 × 10^−3^	29.4	3	3/4 ^2^	Likely Pathogenic/Pathogenic
												
*PMS2 ^3^*	172	12	0.021%	No dbSNP	7	6043400	T/C	.	24.9	3	6	-
												
*BRCA1*	344	17	0.071%	Not found	-	-	-	-	-	-	-	-
												
*HOXB13 ^3^*	62	0		Not found	-	-	-	-	-	-	-	-

LoF: loss-of-function, stop gain/loss, splice-site, and frameshift indel variants; No: number; NFE: Non-Finnish European; US: uncertain significance; Conservational Scores: Genomic Evolutionary Rate Profiling (GERP), PhastCons, and Phylogenetic P-value (PhyloP); inclusion cutoff ≥ 2/3; Prediction Tools: Sorting Intolerant from Tolerant (SIFT), Polymorphism Phenotyping version-2 (PolyPhen-2) HDIV (HumDiv), PolyPhen-v2 HVAR (HumVar), Log ratio test (LRT), MutationTaster, Mutation Assessor, Functional Analysis Through Hidden Markov Models (FATHMM), MetaSVM, MetaLR, Protein Variation Effect Analyzer (PROVEAN); inclusion cutoff ≥ 6/10; ^1^ probability of carrying pathogenic/likely pathogenic non-synonymous and loss of function (LoF) variants in the ExAC population; ^2^ data from 4 prediction tools available; ^3^ the high-risk status of PMS2 and HOXB13 is under discussion.

## Supplementary Materials

The following are available online at https://www.mdpi.com/2072-6694/12/10/2770/s1, Figure S1: Distribution of the number of GWAS-identified risk alleles in the cancer free families (CFFs) and the 1000 Genomes population for the (a) breast cancer, (b) colorectal cancer, and (c) prostate cancer risk loci.
