# Supplementary material for: Cancer Predisposition Genes in Cancer-Free Families
Source: Cancers (Basel). 2020 Sep 27;12(10):2770. doi: 10.3390/cancers12102770 (PMC7600438; doi:10.3390/cancers12102770)
Supplement: Supplementary file 1 [file cancers-12-02770-s001.pdf]

Supplementary Materials: Cancer Predisposition Genes in Cancer-Free Families

Guoqiao Zheng, Calogerina Catalano, Obul Reddy Bandapalli, Nagarajan Paramasivam, Subhayan Chattopadhyay, Matthias Schlesner, Rolf Sijmons, Akseli Hemminki, Dagmara Dymerska, Jan Lubinski, Kari Hemminki and Asta Försti

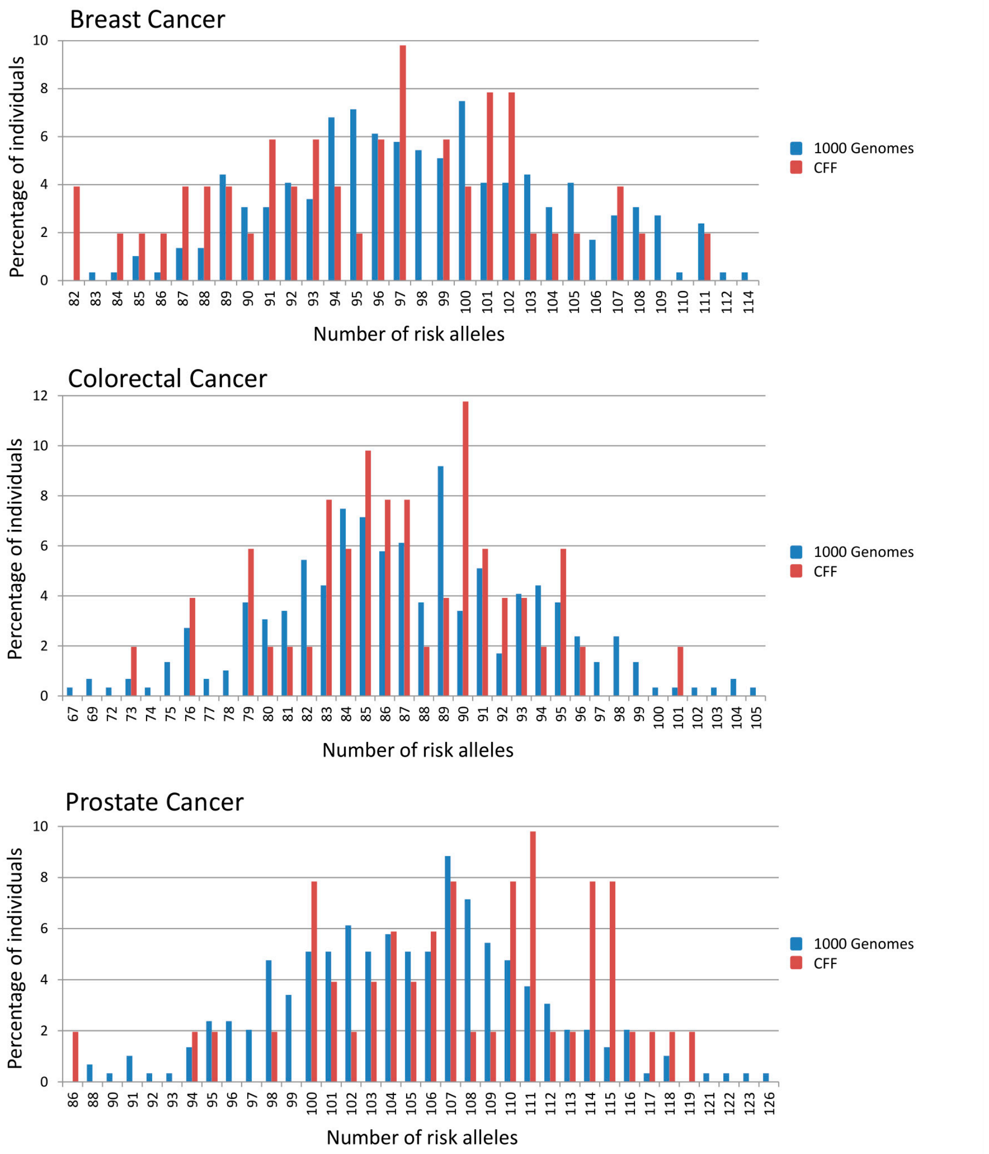

Figure S1. Distribution of the number of GWAS-identified risk alleles in the cancer free families (CFFs) and the 1000 Genomes population for the (a) breast cancer, (b) colorectal cancer, and (c) prostate cancer risk loci.

© 2020 by the authors. Licensee MDPI, Basel, Switzerland. This article is an open access article distributed under the terms and conditions of the Creative Commons Attribution (CC BY) license (http://creativecommons.org/licenses/by/4.0/).
